# Leptospirosis and the Environment: A Review and Future Directions

**DOI:** 10.3390/pathogens12091167

**Published:** 2023-09-16

**Authors:** Elizabeth A. Bradley, Graeme Lockaby

**Affiliations:** College of Forestry, Wildlife, and Environment, Auburn University, Auburn, AL 36849, USA

**Keywords:** leptospirosis, *Leptospira*, environmental zoonoses, neglected tropical diseases, one health

## Abstract

Leptospirosis is a zoonotic disease of global importance with significant morbidity and mortality. However, the disease is frequently overlooked and underdiagnosed, leading to uncertainty of the true scale and severity of the disease. A neglected tropical disease, leptospirosis disproportionately impacts disadvantaged socioeconomic communities most vulnerable to outbreaks of zoonotic disease, due to contact with infectious animals and contaminated soils and waters. With growing evidence that *Leptospira* survives, persists, and reproduces in the environment, this paper reviews the current understanding of the pathogen in the environment and highlights the unknowns that are most important for future study. Through a systematic Boolean review of the literature, our study finds that detailed field-based study of *Leptospira* prevalence, survival, and transmission in natural waters and soils is lacking from the current literature. This review identified a strong need for assessment of physical characteristics and biogeochemical processes that support long-term viability of *Leptospira* in the environment followed by epidemiological assessment of the transmission and movement of the same strains of *Leptospira* in the present wildlife and livestock as the first steps in improving our understanding of the environmental stage of the leptospirosis transmission cycle.

## 1. Introduction

### 1.1. Overview and Biology of Leptospirosis

Leptospirosis is a global zoonotic disease estimated to cause around 1 million cases and 60,000 deaths annually [1]. Caused by infection with bacteria from the genus *Leptospira*, leptospirosis presents a variety of symptomology ranging from asymptomatic to mild febrile illness to severe acute infection resulting in organ failure and death [1,2,3,4,5]. Additionally, as many as 30% of leptospirosis cases result in long-term health impacts [4,6]. There are many serovars and strains of *Leptospira,* with those that are pathogenic being of primary concern [2,3,5,7]. *Leptospira* reproduction usually occurs in the renal tubules of infected mammals [2,4,5,8]. *Leptospira* are excreted, via urine, into the environment where they may infect other organisms [1,8]. *Leptospira* infections usually occur through abrasions or wounds in skin or through contact with mucosa [5]. *Leptospira* can be transmitted directly to humans through the handling of infected animals, making leptospirosis an occupational hazard for people that regularly handle animals, such as livestock producers, abattoirs, veterinarians, hunters and game managers, animal control workers, and scientists [1,2,3,4,7,9,10,11]. As the local prevalence of leptospirosis and the frequency of exposure to *Leptospira* determines the risk of infection for an individual, individuals in regions with increased workplace procedures and protections such as surveillance, diagnostic testing and treatment of infected animals, and access to personal protective equipment—such as gloves, goggles, and boots—are less likely to contract leptospirosis [1,3,4,7,11,12,13]. More commonly, leptospirosis is contracted indirectly through contact with contaminated water or soil [2,4,5,7,8,11]. As a result, leptospirosis remains an occupational hazard for individuals that work closely with soil and water systems, such as sewage and waste workers, construction, military, aquaculture workers, and farmers [1,4,7,8,9,11,12,14,15,16,17,18]. Leptospirosis is also a growing environmental hazard for outdoor recreationalists—such as kayakers, swimmers, fishermen, etc. [7,12,14,15,16,19,20,21,22,23,24,25,26,27,28,29,30,31,32,33]. As outdoor recreation continues to grow, diagnosis of leptospirosis is expected to grow comparably.

In addition to being an occupational and environmental hazard, leptospirosis has been designated by the U.S. Centers for Disease Control (CDC) as a neglected tropical disease (NTD), characterized by its impacts on vulnerable populations and the diversity of epidemiological settings the disease is found in [3,11,34]. Due to the disproportionate impacts of the disease, epidemiological efforts to control leptospirosis are best focused on the most vulnerable populations, including socioeconomically disadvantaged populations in high-density urban environments and those in closest contact with infected animals, such as rural farmers [1,4,11,16,35]. Studies across the globe find significantly increased cases of leptospirosis and disproportionate transmission rates of *Leptospira* in communities of lower socioeconomic status due, in no small part, to inadequate sanitation infrastructure and/or lack of enforcement of safe drinking water standards [4,16,35,36,37]. Increased local prevalence of the disease and frequency of exposure determines the transmission rates, so these findings result from greater local prevalence of the disease-causing pathogen and greater frequency of exposure in socioeconomically disadvantaged communities. The frequency of exposure to the pathogen and the local prevalence of the disease is largely due to environmental conditions, which we will discuss in further detail later in the review. However, it is important to note that many of the environmental conditions associated with lower socioeconomic status and rates of leptospirosis result from social inequality. These conditions include contact with sewer water, trash accumulation, and reduced rates of governmental sanitation interventions, such as failure to control rodent populations, maintain municipal trash removal and sanitary sewers, and provide universal access to potable drinking water [4,38]. In addition to the conditions associated with leptospirosis that result from socioeconomic inequality, the burden of leptospirosis on socioeconomically disadvantaged communities further facilitates cycles of poverty due to the expense of medical treatment, lost wages while ill, and persistent long-term health effects of the disease. Thus, leptospirosis can serve as both the result of and the cause of poverty [3,34,39]. The burden of leptospirosis and its role in increasing poverty is only expected to grow through increasing urbanization, which often outpaces the establishment of governmental sanitation interventions in socioeconomically disadvantaged communities [40,41]. Rapidly increasing urbanization will likely increase the conditions associated with the disease, as will be further discussed later in this review.

### 1.2. Current Status of Leptospirosis

Currently, leptospirosis is a disease of significant concern. The World Health Organization (WHO) estimates that globally the endemic human leptospirosis rate is 5 cases per 100,000 people annually and the epidemic human leptospirosis rate is 14 cases per 100,000 people annually [42]. However, more recent estimates suggest that leptospirosis is “among the leading zoonotic causes of morbidity and mortality” and causes around 1,000,000 cases annually [1]. As shown by the conflicting estimates, the current global burden of leptospirosis is difficult to determine.

The current challenges surrounding obtaining a global estimate of leptospirosis morbidity and mortality largely stem from the compounding issues that arise when critical components of a disease’s lifecycle are not understood. For example, in an effort to clarify the global issue of leptospirosis, the World Health Organization (WHO) leads an initiative seeking to improve global estimates of morbidity and mortality of the disease through efforts to address the lack of surveillance, adequate diagnostic tests, and prompt treatment as well as the rates of misdiagnosis and underdiagnosis of the disease [1,11,16,43,44,45,46,47,48]. The issue of misdiagnoses and underdiagnoses results from and are due to, in part, to a lack of understanding on the prevalence of leptospirosis in a given region, as this hinders physicians from knowing whether leptospirosis should be tested for [1,44,49]. Additionally, of concern is who (individuals, health insurance, or public health initiatives) is responsible for the costs of testing for and treatment of the disease. The ability to measure the prevalence of a disease usually depends on standard surveillance of livestock, wildlife, and human infection. In the United States, these are federally mandated and funded disease surveillance programs that include screening, sampling, testing, treatment, and testing components with required reporting. Outside of the United States, the WHO makes recommendations for testing and reporting within member nations. Depending on the level of urgency and committed funds, some testing and reporting of NTDs, such as leptospirosis, occurs at a member nation’s laboratories or at regional WHO reference laboratories. Lower priority diseases may only be available through private laboratories. The major limitation of such surveillance approaches is that the priority ranking and allocation of funding is based on national or regional prevalence and transmission rates, which can be greatly masked or suppressed by inadequate testing. Additionally, the standard methods of disease surveillance are inadequate for leptospirosis as there is growing evidence supporting significant *Leptospira* transmission in the environment [1]. However, very little is known about the survival, persistence, and reproduction of *Leptospira* in the environment or about the routes of transmission of *Leptospira* within and from the environment. This makes efforts to improve our understanding of the environmental stage of the leptospirosis transmission cycle essential to understanding global morbidity and mortality rates. As our knowledge of where the disease is most prevalent, has the most severe impact, and the routes of transmission that facilitate the persistence and spread of the disease in such communities increases, our ability to respond to and reduce the disproportionate impacts of leptospirosis will improve.

While already imperative, addressing the disproportionate impacts of leptospirosis will become increasingly more crucial as climate change increases the frequency and severity of severe weather events and the rapidly changing climate increases the burdens on socioeconomically disadvantaged communities in tropical nations [4]. Closely associated with severe weather events, leptospirosis outbreaks are common across the world following floods, storms, and other mass precipitation events [8,50,51,52,53]. As such, it is crucial that efforts are made to better understand the environmental stage of the disease cycle so that better disease surveillance, diagnostics, and disease interventions can be implemented.

### 1.3. Objectives of this Review

In response to leptospirosis’ status as a neglected tropical disease and the growing need to understand the environmental stage of the disease’s transmission cycle, this review examines the literature, synthesizes the current understanding, and highlights future directions for further research. We will cover leptospirosis in the three major components of the environment: water, soil, and the organisms that facilitate the movement and transmission of the pathogen in the environment. In addition, we will also review how these components may differ along the urban–rural gradient. Due to the global extent of *Leptospira* and the impacts that leptospirosis has on human and animal health worldwide, our review will follow a broad, global discussion of the literature and findings.

## 2. Materials and Methods

To compile this review, we used numerous literature bases, including Web of Science, BioOne Complete, Elesvier, and several others (see list of databases searched at hhtps://www.lib.auburn.edu/find/bytitle.php, accessed from 1 September 2022). Within these literature bases, we conducted a Boolean search including the combination of the following search terms: [Leptospir* AND (transmission OR water OR soil OR water OR wildlife OR environment OR mortality OR ecology OR survivorship OR urban OR rural)]. No age range was selected. Potential publications were reviewed by the authors to determine those that were comparable in study subject, study focus, and methodological approach; this was performed to focus on leptospirosis in context of the environment and remove strictly papers unrelated to *Leptospira* in the environment. If the papers did not contain a focus on the environment or contain a focus on the given search terms, then they were excluded. Then the results of these studies were synthesized for this review, whether their findings contrasted or supported each other. A total of 1,463 publications were found using our literature bases for use in this review. Once these publications were collected, more than 500 duplicate publications, more than 300 out-of-scope publications, and more than 300 publications without full-text were removed from our list of reviewable manuscripts. Ultimately, ~220 publications were selected to be cited in this review. Any additional publications cited in this review were used because they covered information pertaining to topics covered in the review or they otherwise improved this review with their content. These papers were largely examples of environmental studies that have been performed on other zoonotic pathogens, such as *Escherichia coli*, but have yet to be conducted for *Leptospira*.

## 3. Leptospirosis in the Environment: Water

While leptospirosis is reliant on the continuous cycle of transmission of *Leptospira* from infected to uninfected animals, there is growing evidence that there is long-term survival and viability of the pathogen in the environmental stage of the transmission cycle [8,54,55,56]. This is important because the opportunity for transmission of the pathogen increases the longer that *Leptospira* survives and remains a persistent feature in the environment. With the recent laboratory-based discovery of *Leptospira* replication within waterlogged soil emphasizing the critical role that water serves for *Leptospira* outside of animal hosts, it is more apparent than ever that future research should be conducted to better understand the survival, persistence, and reproduction of *Leptospira* in waterways [54,57].

### 3.1. Current Understanding

Due to the frequent linkage of outbreaks with exposure to contaminated waters, leptospirosis has often been discussed as a water-borne disease [3,58]. Additionally, higher rates of leptospirosis are linked to regions with greater percentages of riparian habitat and surface waters [59]. While not strictly water-borne, outbreaks of leptospirosis following floods, storms, and other mass precipitation events have been documented across the world [8,50,51,52,53].

Outbreaks of leptospirosis following floods, storms, and other mass precipitation events originally linked the disease to environmental waters; however, many detection techniques have developed over the decades to facilitate active surveillance of the disease in environmental waters. The most promising techniques for the study of pathogenic *Leptospira* in waters in the environment are quantitative real-time polymerase chain reaction (PCR) tests that target only pathogenic *Leptospira* spp. [60,61]. There are key genetic features that appear to be conserved in pathogenic *Leptospira*, making detection techniques that target these retained gene patterns the preferential method as they allow for discussion of public health implications and comparison to animal and human studies in the area (provided that they used serological and molecular diagnostic techniques that target the same pathogenic gene patterns) [55,60,62]. Optimization in recent years of these methods for the quantification of pathogenic *Leptospira* in environmental waters has accelerated studies of environmental *Leptospira* and tracing of human leptospirosis to environmentally present *Leptospira* in regions across the world [63,64,64,65,66]. Additionally, these advances are accelerating the rate of further optimization of methods, which improves the testing in other conditions (such as soil) and the results available for modeling [67,68].

One of the most critical and currently perplexing issues in the study of leptospirosis and the survival of *Leptospira* in environmental waters is the range of conditions in which the pathogen is present and surviving [8]. Survivorship or hardiness of *Leptospira* in water covers a wide range of conditions. For example, *Leptospira*, both pathogenic and not, appear capable of surviving long periods under both low and high temperature ranges [8,69,70,71]. They also appear to survive in a range of pH levels, dependent on the strain and the presence of other microbiota, such as bacteria and fungi [8,69,70,71,72,73,74]. Additionally, pathogenic *Leptospira* seem capable of surviving in waters with low nutrients, which indicates that they are likely present in streams and waterways across a far greater range of physiographic and hydrologic conditions than previously expected [8,69,70]. Not only do they appear capable of surviving in low nutrient conditions, but they also appear to retain virulence over long periods of starvation [75]. However, in our review of the literature, we found an extremely limited number of field-based environmental studies attempting to evaluate the survival of *Leptospira* in the environment [55,56,76]. Due to the lack of studies, we have yet to identify results about environmental conditions that facilitate the survival of *Leptospira* in surface waters [Table 1].

### 3.2. Future Directions

As we have previously mentioned, there are many unknowns about *Leptospira* in environmental waters. Through our review, we have determined several key areas in need of targeted study. Primarily, there is a substantial need to focus on gathering information on the conditions of survival, persistence, and potential reproduction of pathogenic *Leptospira* in natural waters. It is essential that these studies be conducted in field conditions so that the variability of natural conditions informs the finding of the probable capabilities of *Leptospira*. Additionally, there is a substantial body of research in the literature exploring methods of detection and survivability of *Leptospira* in water sources, but there is still a substantial need to apply these methods in natural conditions to determine the survivorship of viable and infectious *Leptospira* [8,60]. Studies should use natural systems, target viable and infectious pathogenic *Leptospira*, and consider the complex processes and systems that drive the results of the study. With these specific considerations in place, we recommend the following additional future directions:

Firstly, lack of surveillance, which results from, and is at least partially attributable to, the costs and challenges with detection methods, limits our current understanding of the role of environmental waters on the transmission cycle of leptospirosis. One of the limitations for the measurement of *Leptospira* in the environment is that methods are often optimized for sampling under certain conditions over others and, thus, result in variability between conditions that hinder interpretability of results. For example, sampling of environmental water sources by methods that incorporate filtration can be complicated by the clogging of the sample filters as a result of physiochemical characteristics of the water such as increased concentrations of suspended solids, dissolved organic carbon, and other dissolved nutrients [63,67,77,78]. However, alterations to methods and procedures in a recent study provide evidence that variability in quantification of *Leptospira* collected from different water sources—such as ponds, streams, and sewage waters—can be reduced and accounted for [63]. Another limitation for the surveillance of *Leptospira* in the environment results from the complex and time-consuming nature of *Leptospira* isolation. However, methods for the identification of *Leptospira* from environmental sampling have been improved in recent years resulting in reduced laboratory time constraints [63,67,78,79]. One such method involves the enrichment culturing followed by PCR targeting the gene *lipL32*, which allows researchers to confirm the presence of pathogenic *Leptospira* [78]. Other studies target the 16s rRNA gene described by Schneider et al. 2018 [8,67,80]. The latest studies are beginning to evaluate the use of next-generation sampling—such as Oxford Nanopore Technologies—and mass spectrometry—such as matrix-assisted laser desorption ionization time-of-flight mass spectrometry using MALDI Biotyper Systems—for the sequencing of samples directly [78]. These improvements in methodology have resulted in studies reporting the identification of novel *Leptospira* from the environment, with one such study resulting in the identification of twelve novel species [79,81]. In addition to allowing for direct sequencing of environmental samples, the improvements to methods in recent years have created promising solutions to one of the most critical distinctions when studying *Leptospira* in the environment: whether the measured *Leptospira* are pathogenic or not. One of the final limitations to environmental surveillance of *Leptospira* is the difficulties with identifying whether measured *Leptospira* are virulent or not. As the point of conducting environmental studies of *Leptospira* transmission is to determine whether the environment is serving as a reservoir and contributing to the transmission of the disease to uninfected organisms, measures of the infectability and viability of environmental *Leptospira* are critical. However, recent applications of methods and techniques, such as culturing/optical densities or viability PCR, are creating promising solutions to this issue [55,74,82]. By utilizing approaches that focus on the viability of pathogenic strains of *Leptospira* from environmental samples, studies are beginning to identify potential factors that facilitate the survival of *Leptospira* in surface waters, such as calcium, iron, and pH [74]. As such, the advancements in methods and approaches in the past decade are rapidly addressing many of the current limitations of environmental surveillance of *Leptospira* and will serve a critical role in addressing the unknowns of its environmental persistence, survival, and transmission.

Secondly, understanding the driving mechanisms behind the persistence, survival, and transmission of *Leptospira* through water has proven elusive [54]. The characteristics of the waters in question, as well as the biogeochemical and hydrologic processes these characteristics result from, have significant influences on these driving mechanisms. As mentioned previously, pathogenic *Leptospira* survives for long periods in a range of different pH levels, nutrient levels, and temperatures. Such water quality metrics originate from biogeochemical and hydrologic processes associated with the stream environment. Despite this, few studies exist that consider such factors. As a result, there is a severe scarcity of information on the impacts of basic water quality metrics, such as nitrate levels, on the abundance and survivorship of *Leptospira*. Additionally, the standard water quality metrics that studies are including have the potential of being misinterpreted without additional biogeochemical and hydrologic processes being considered. For example, reports suggest that *Leptospira* concentrations increase with the turbidity of streams, a stream characteristic that often rises following large rainfall events and flooding events [8,83]. One theory behind this observation is the capacity of elevated turbidity levels to shield bacteria from UV irradiation through increased levels of suspended solids, which is a direct threat to *Leptospira* persistence and survival in surface waters in tropical regions [84]. The mechanism of suspended solids shielding pathogens from UV radiation has been demonstrated with fecal indicator species in natural conditions [85]. However, as a stream characteristic and water quality metric, turbidity reflects cloudiness in water; turbidity cannot be used directly as a measure of suspended sediments since cloudiness may stem from other sources including tannins. This is just one example of the potential for misinterpretation without attention being paid to specific processes and stream characteristics that may influence what is being observed. Additionally, this study did not identify a *Leptospira*-specific study of this mechanism and, thus, it remains unknown in the literature.

Another future direction is more attention to the intrinsic properties and behaviors of *Leptospira* and how that may facilitate survival, persistence, and reproduction in environmental waters. One such example is the ability of some strains, particularly pathogenic strains, to metabolize urea, which may improve the ability to survive for longer periods in smaller waterways particularly those that are nutrient-poor [86,87,88]. However, we are unaware of studies that attempt to assess this in natural waters so future research is needed. Additionally, part of the survival of *Leptospira* in freshwater may be the result of interactions with environmental bacteria and the formation of cellular aggregations called biofilms [89,90,91,92]. These aggregations are held together through an extracellular matrix made of nutrients such as nucleic acids, proteins, lipids, and polysaccharides, which creates a more hospitable environment for the pathogen alongside other microbial species that may be present in the environment [89,93]. Future studies into the survival and viability of *Leptospira* in the environment should focus on biofilms including microbial relationships and conditions found in communities of *Leptospira* in streams. Existing methods found in water microcosms may be improved by the methods and frameworks used in the extraction and quantification of biofilms in medical contexts [94,95]. If these methods prove useful, then attempts to use them in field studies would be the next steps for assessing the impacts of these intrinsic properties of *Leptospira* and their interactions with other bacteria.

Finally, in addition to future studies considering the biogeochemical processes and hydrologic characteristics of the waters being studied, hydrologic and climatic modeling could be used to improve the risk maps created by socioeconomic and epidemiological modeling that help identify areas most suitable for long-term persistence and transmission of *Leptospira* [37,68,96,97,98,99,100]. These types of models are improved with the addition of new data, so long-term collaborative mapping between experts in different specialties would provide the maps most useful for targeting surveillance, management, and future research in regions where the use of such resources would be most effective. These maps would also facilitate discussion of region-specific trends of environmental persistence and transmission of *Leptospira*, which—due to limited studies to draw from—are currently infeasible to draw conclusions on.

## 4. Leptospirosis in the Environment: Soil

*Leptospira*, both pathogenic and not, have been found in soils globally [8,56,57,62,64,95,101,102,103,104,105,106,107,108,109]. Contact with *Leptospira* in the soil is one of the leading routes of infection with leptospirosis, resulting in outbreaks across the globe [2,3,4,7,9,12]. As mentioned previously, outbreaks of leptospirosis following floods, storms, and other mass precipitation events have been documented globally [8,50,51,52,53]. The leading hypothesis for this phenomenon is that outbreaks of leptospirosis result from the resuspension of the pathogen from the soil, where viable *Leptospira* may persist for extended periods [8,52,53]. However, just as with the current understanding of *Leptospira* in water, very little is currently understood about the growth, survival, and long-term persistence in soils. As new evidence supporting *Leptospira* multiplication in soil suggests, understanding the current knowledge and working to address existing gaps in the literature on *Leptospira* survival, persistence, and reproduction is essential [57].

### 4.1. Current Understanding

As discussed earlier, a general understanding of the risk of leptospirosis has emerged regarding the environmental conditions under which transmission to and subsequent infection of humans may be most susceptible to exposure. A high risk of transmission is associated with tropical settings, major precipitation events such as hurricanes, and contact with wet soils and surface waters that are contaminated by infected mammals and other animals [8,110]. These risk factors are sufficiently well-established to be used in alerts provided by public health agencies, such as the CDC [111]. Similarly, most agree that survival and persistence of *Leptospira* are higher in soils than in surface water, although this could reflect the difficulty of detecting the pathogen after major dilution occurs in surface waters [8,107]. Ultimately, we lack a detailed understanding of why there might be greater Leptospira numbers in soils or what potential mechanisms would be driving such a phenomenon [54].

We are unaware of studies assessing the texture and other physical properties of soils associated with Leptospira survival. However, the texture—portion of sand, silt, and clay-sized particles that comprise the mineral fraction of the soil—and physical properties of soils—such as soil moisture content, pH, organic matter, soil organic carbon, total nitrogen, and the carbon to nitrogen ratio—can have significant impacts on the survival and persistence of zoonotic pathogens and microbial populations in soils [112,113,114]. Additionally, the texture and physical properties of soils in streams and waterways can directly influence Leptospira survival and persistence. Fine sediment material is readily suspended in the water column, while large soil particles, such as sand, usually drop out of the column. This can affect the deposition and resuspension of zoonotic pathogens in natural streams [115,116]. Following major rainfall events, clay sediments tend to be carried easily with sheet flow and add to turbidity as would organic particles. In addition, Leptospira bacteria may adhere to soil particles through adsorption, which is a trait that facilitates stream suspension [8,55]. Clayey soils and organic particles are more active in the adsorption of materials compared to sands since they have more surface area per unit of mass. Consequently, we suggest that soils providing the most protection and the highest likelihood of movement with floodwater would be finer textured clay soils with significant organic matter content. In addition to the capacity of clayey soils to facilitate the movement of water-borne pathogens through their resuspension rates and high runoff potential, clay soils have a greater field capacity than sandier soils and, thus, hold on to more water than other soil textures [117]. The greater capacity of clay soils to hold on to water likely facilitates *Leptospira* survival. Statistical support for this hypothesis is demonstrated within studies from America Samoa and the Netherlands, which both identified a significant positive association between cases of leptospirosis and clay soils [105,118]. Although more research into this potential association is needed, their findings support the widespread agreement that wetter soils, i.e., greater than 20% moisture, and waterlogged soils serve as environmental reservoirs for Leptospira [54,57]. However, it is unclear from some of these studies whether the soils used in the studies were truly anoxic or not. If these soils were truly under anoxic conditions, then not only would the transformations of nutrients—such as nitrogen—occur following anaerobic mechanisms but also by anaerobic bacteria species. An example of this is clearly demonstrated with the transformation of nitrogen in anaerobic soils conditions through anammox or through denitrification, both of which are processes led by species from different genera of prokaryotes [117]. Thus, depending on the conditions the soils being studied are exposed to, there could be significantly different microbial communities and biofilms, which may greatly influence the survival and persistence of Leptospira. Additionally, if Leptospira bacteria are found to be surviving and persisting in truly anoxic conditions, then it may be time to reclassify Leptospira from obligate aerobes to facultative anaerobes [119]. This is an important distinction, because if the bacterium is facultatively anaerobic, then it may be persistent across a wider range of soil conditions than previously thought.

Additionally, there is little information, much of which is conflicting, regarding the range of soil physiochemical properties that are most conducive to Leptospira persistence [54,104]. Some reports indicate that neutral to high pH conditions (i.e., 7-8) are most suitable [54,55]. However, this assertion conflicts with positive correlations between Leptospira persistence and concentrations of Fe, Mn, and Cu, elements that increase in concentration under acidic pH conditions, and negative correlations with Ca, which increases with higher pH conditions [104]. As in waters, the ability of Leptospira to survive and persist under such a range of often adverse soil conditions may be the result of biofilms, which create more suitable conditions for bacterial survival in soil, as has been seen with other zoonotic pathogens such as *Escherichia coli* [120]. Early study into this suggests that Leptospira are capable of producing these biofilms in soils and with several other species of bacteria found in the environment [93,94,121,122].

### 4.2. Future Directions

Much of the current literature gaps involving leptospirosis and soils exist due to a lack of field study and research on soil characteristics and dynamics. We know very little about what strains of *Leptospira* are present in the environment. As strains of *Leptospira* from across the genus are present in soils, future studies should seek to assess the full diversity of *Leptospira* that persist, survive, and reproduce in soils. We expect great diversity of *Leptospira* in the soil as 12 new species were identified in a single study [79]. Assessment of *Leptospira* in soil should not be confined to searches for strains found in the traditionally ‘pathogenic’ subclade P1, as strains from both subclades are linked with human cases of leptospirosis and survival in soil. For example, both *L. interrogans* and *L. licerasiae* are linked to human cases of leptospirosis and survival in soil environments [95,123].

In addition to identifying the strains of *Leptospira* that are present in the environment, future study is needed to determine the soil conditions required for the persistence, survival, and reproduction of each strain. Our first recommendation is that care must be taken not to neglect describing and measuring the soils being used within future studies. As soil microbiologists can attest to, the biogeochemical processes of soils are complex and dynamic systems that are certain to greatly influence the strains of *Leptospira* that survive, persist, and reproduce in the soil. Future study is also needed to assess the conditions that facilitate the virality of *Leptospira* in the environment, as this is a key question of epidemiological concern. Knowing so little about the persistence, survivorship, and reproduction of individual *Leptospira* spp., we cannot begin to determine or speculate whether the required conditions for each are common among the genus or are specific to each strain. As a result, we highly recommend that future studies include reference to the soil survey for the study site, which contains the soil classification, general information about the geology, topography, and climate of the area, and list the types and volumes of soils in the area. Such surveys within the United States can be accessed online via the National Cooperative Soil Survey. Outside of the United States, local soil scientists should be contacted for assistance accessing local soil surveys. In addition to referencing the soil survey for the classification of the studied soil, an assessment of the physiochemical properties of the soils is essential. We recommend that such assessments include physical and chemical indicators of soil health, such as nutrient levels, bulk density, soil moisture, and texture.

While there is evidence that *Leptospira* can interact with other soil biota and create biofilms that may improve their persistence and survival in adverse soil conditions, there is little else known about the interaction of *Leptospira* with other soil microbiota and the role that these interactions (direct or otherwise) may have on the pathogen’s survival [91,121,122]. With so little known about the topic, any future studies would be widely beneficial to beginning to form hypotheses on the long-term survival, persistence, and reproduction of *Leptospira*. In addition, as was emphasized in the discussion of *Leptospira* and environmental waters, it is imperative that future study be conducted in natural environments. Lab-based studies are unlikely to include the tremendous variability in biotic, soil, and climatic conditions that are known to influence soil microbiota. As a result, the findings of such lab-based studies are unlikely to represent the probable survival, persistence, or reproduction of *Leptospira* in natural conditions.

## 5. Leptospirosis Enzootic Persistence in the Environment: Organisms of Concern

Based on our current understanding of the lifecycle of *Leptospira*, the enzootic persistence of the pathogen in water and soil is reliant on the continual excretion of the pathogen by infected animals. Leptospirosis affects many animal species, both domestic and wild [1,8,124]. Despite most mammals serving as competent hosts and vectors of *Leptospira*, very little is known about the pathogen load excreted by different species or the role that specific species play in establishing and maintaining environmental sources of the disease [54]. Additionally, as impetus for a universal vaccine does not appear to be available soon, and with international trade of domestic and wild animals likely to increase and contribute to the global spread of leptospirosis, we have outlined some of the current knowledge surrounding the disease in native and introduced species, with particular attention paid to species with the most potential or known capacity to spread the disease [125,126].

### 5.1. Domestic

Domestic, non-native species are often the dominant reservoir of *Leptospira* in rural regions and urban regions [4,127]. Some domestic species are extremely widespread, transient in nature, and continue to shed *Leptospira* persistently in their urine long after their initial infection has subsided [127]. For this review, four such domestic animal groups (rats, dogs, pigs, and cattle) are used to illustrate the capacity that domestic species serve in the persistence, spread, and levels of *Leptospira* in the environment.

The black rat, *Rattus rattus*, and the brown rat, *Rattus norvegicus*, are largely considered the most important sources of leptospirosis [3,4,12,20,54,59,127,128,129,130,131,132]. Introduced globally, the black and brown rat continues to thrive alongside human populations in both urban and rural settings. Rats are asymptomatic carriers (hosts) for many strains of pathogenic *Leptospira*, are commensal hosts that are persistently infected, and shed the pathogen at levels that exceed many other competent hosts for the disease (7 × 10^7^ per day) [132,133,134]. Linked historically to human outbreaks of leptospirosis across the globe, rats continue to play a significant role in the transmission of the disease in urban environments, particularly in low-income communities [128,129,130,131,134,135,136,137,138,139,140]. However, the role of rats in the disease cycle of leptospirosis is not limited to urban settings, as they can be quite prevalent in rural regions as well [134,141]. Perhaps the best studied of the animals associated with leptospirosis, there appear to be critical gaps in the literature remaining to study about the role that rats play in the persistence and load of *Leptospira* in the environment. Studies that seek to link strains of *Leptospira* in the environment with their animal host will encounter rats due to their global distribution, high population densities, and competency to spread the pathogen.

Domestic dogs are also highly competent hosts and participate in the spread of the pathogen globally [127,142,143,144]. Despite spending so much time in close proximity to humans, dogs do not seem to contribute to outbreaks in humans and instead seem to impact the disease cycle through their contribution to environmental contamination [145,146]. The majority of strains of canine leptospirosis cases are now associated with the environment, largely from contaminated waters [142]. Once infected, domestic dogs can spread the *Leptospira* (via urinary excretion) across large distances as a result of their highly mobile behavior (averaging 5 km per day and up to 330 km during human-mediated activity in one study) [147]. Not only are dogs able to spread *Leptospira* long distances, but they also excrete large numbers of the organism in their urine (1.6 × 10^5^ per day) [132]. In addition to the long distances traveled by dogs and the large number of the pathogen excreted, shedding of *Leptospira* has been documented to last anywhere from four to six weeks—and in some cases for several years [148]. However, despite their capacity to spread *Leptospira* long distances and in great quantity over a substantial time period, little to no research has been conducted to evaluate whether leptospirosis outbreaks in wildlife are the result of canine-shed *Leptospira*. This is a key gap in the literature because vaccinations are available for canines and could be used to reduce the environmental load of *Leptospira*. As mass vaccination efforts are resource and effort-intensive, it is important that the capacity of dogs to contribute to wildlife leptospirosis outbreaks be assessed accurately and quantitatively prior to such efforts being made. Additionally, as indicated in the great spatial range facilitated by the movement of dogs by humans, dogs—particularly unowned, unconfined, or free-roaming dogs—may serve a critical role in the transport of infectious *Leptospira* along the urban–rural gradient.

Leptospirosis is a well-established disease of the ungulate species, *Sus scrofa*. Pigs, domestic or wild, can spread the pathogen to humans directly as frequent infections in hunters and pork producers illustrate [149,150,151,152,153,154]. Pigs may be significantly impacted by leptospirosis, causing reproductive failures including infertility, premature and stillbirth, and fetal death [154,155,156]. Additionally, infected pigs may have persistent renal infections and persistent shedding of *Leptospira*. Due to the unique foraging behavior of pigs called rooting, which involves the turning of soil with their snouts, they are exposed to *Leptospira* in the soil that has direct contact with the mucosa in their snouts. Another of their behaviors, wallowing in the soil, may also play a role in the transmission of *Leptospira,* as other pigs and many other wildlife species drink from these wallows [157]. A study evaluating the prevalence of *Leptospira* in pig wallows and molecular tracing of the disease in wildlife from the area would reveal whether these wallows are playing a role in the transmission of leptospirosis in the environment. In addition, their natural social behavior encourages and provides close contact between potentially infected and non-infected individuals in wild environments as well as in domestic production [158]. As such, pigs are uniquely well-suited for contributing to both the introduction and persistence of *Leptospira* in the environment. However, very few studies have investigated the role that pigs have in the environmental transmission of *Leptospira*. Most existing studies have focused on the prevalence of leptospirosis in pigs and the capacity of wild pigs to serve as the reservoir for *Leptospira* to transmit to domestic pigs and livestock [159,160,161,162]. The potential for wild pigs to spread the disease is of significant concern throughout their range, both native and non-native [150,163,164,165,166,167,168,169,170,171]. This is due to a striking increase in feral pig populations across the globe and how common zoonotic pathogens are in the species, with 87% of swine pathogens listed by the World Organization for Animal Health being zoonotic, impacting humans, livestock, and wildlife species [163]. Their foraging behavior puts them in close contact with soils, and pigs spend significant time in and around waterways due to their inability to effectively thermoregulate. As a result, they contaminate waterways directly through their time in and around waters and indirectly through their waste [172]. Although we are unaware of studies directly evaluating soil and water contamination with *Leptospira* resulting from runoff and spills from swine sewage lagoons from swine-rearing facilities, studies indicate that swine pathogens can contaminate waters and soils surrounding the swine industry [173]. Therefore, future studies evaluating pig contamination of waters and soils are needed. Additionally, despite their tremendous potential for disease transmission, we were unable to find a study evaluating the levels of *Leptospira* excreted by pigs in their urine.

Due to the economic burden of the disease on livestock production, leptospirosis in cattle has long been studied [174,175]. Much of this economic impact results from reproductive failures including infertility, premature and stillbirth, and fetal death [159,176]. In addition to the economic impacts, leptospirosis threatens humans through direct transmission of *Leptospira* from cattle [4,16,174,177,178]. Due to management strategies such as allowing unrestricted access to natural water sources, cattle are infected by and contaminate surface waters with *Leptospira* [178,179]. The unrestricted access to surface water also puts cattle in close contact with potentially infected domestic species such as pigs or wildlife species. As discussed, one of the major concerns about wild pigs is their potential ability to spread leptospirosis to cattle. We are unaware of a study linking leptospirosis infection in cattle with strains of *Leptospira* found in nearby wildlife species, such as wild pigs. Future studies should evaluate this, as direct evidence may support modification of the management of livestock in regions with endemic or highly prevalent *Leptospira* in the environment. Other livestock management strategies such as large herd sizes, the introduction of new cattle to existing herds, and keeping pets on the farm all result in a greater risk of leptospirosis among the herd [180]. Additionally, cattle are highly capable of shedding large amounts of *Leptospira* (6.3 × 10^8^ per day) [132]. Fortunately, considering the high rates of *Leptospira* shedding found in cattle, there are highly effective vaccinations available for cattle [181]. However, one consideration of leptospirosis vaccination in cattle is that it is generally only effective for about a year and livestock producers need to continuously vaccinate their herds to prevent the transmission of the disease in their herds. Additionally, evidence suggests that the available vaccines only protect against selected significantly pathogenic/commercially important strains of *Leptospira* [182].

### 5.2. Wildlife

Generally, large herbivores and small mammals are considered to be the most important wildlife species of concern for transmission and enzootic persistence of leptospirosis [8]. However, some evidence suggests that large carnivores such as lynx and wolf species are exposed to leptospirosis frequently—likely in the small rodents and other mammalian prey that they consume [183,184,185]. Wild rodents, including large rodents such as capybara (*Hydrochoerus hydrochaeris*), beavers (*Castor fiber* and *Castor canadensis*), and various species of smaller rodents such as squirrels and mice, are also common wildlife species that carry leptospirosis [186,187,188,189,190,191,192,193,194,195,196,197,198,199,200,201]. Leptospirosis can be extremely common among small mammal populations in an environment [185,186,202], as shown in one such study that reveals 62.4% of small mammals tested carried *Leptospira* [203]. However, as previously discussed, it is difficult to begin to speculate on their impacts on environmental *Leptospira* without further research assessing the shedding rate and urine loads of *Leptospira* in small mammals. It is also clear from the existing literature that future studies must consider the movement of leptospirosis among wildlife species in a broader context with specific attention to what species the disease routinely transmits to and from [204].

While primarily a pathogen associated with mammalian hosts, evidence exists of pathogenic and non-pathogenic *Leptospira* infection in many species of herpetofauna (amphibians and non-avian reptiles) [110,205,206,207,208,209,210,211,212,213,214,215,216,217,218]. Human leptospirosis is known to occur with the handling of infected herpetofauna species, particularly during interactions with captive species [211,212,219]. Many of the herpetofauna species that have tested positive for infection with *Leptospira*, particularly turtle species, spend significant time in and around waterways where they may be contributing to the transmission of zoonotic pathogenic *Leptospira* spp. such as *Leptospira interrogans* [220]. In tropical forests, ground-dwelling herpetofauna, such as some species of snakes, may contaminate the leaf litter and soil, potentially infecting small ground-dwelling mammals. As mammalian species are best known as hosts of leptospirosis, they may be more competent reservoirs and hosts of the pathogen. This could mean that herpetofauna transmits the pathogen to species that are more significant in the disease transmission cycle. Conversely, the opposite may be true. Unfortunately, very little is known about the disease cycle in herpetofauna. A critical gap is the lack of knowledge on the infectious load of *Leptospira* and rate of *Leptospira* excretion in infected species. Future leptospirosis studies in herpetofauna should address the routes of infection, the rate of excretion into the environment, and the role herpetofauna play in the persistence of *Leptospira* in the environment.

Another concern regarding the enzootic persistence of *Leptospira* in the environment is the potential impact on threatened and endangered species. It is well-established that the threat of disease increases as a species becomes more endangered [221]. Endangered wildlife species populations, such as that of the Iberian lynx (*Lynx pardinus*) and the island fox (*Urocyon littoralis*), may be threatened by leptospirosis as well as play a role in the environmental transmission cycle [222,223]. Leptospirosis may also be a threat to threatened marine mammals such as the Amazonian manatee (*Trichechus inunguis*) and the North American manatee (*Trichechus manatus)* [224,225,226,227,228,229]. Much is unknown about the disease in manatees, while other marine mammals suffer clinical disease and reproductive failure with *Leptospira* infection [230]. Therefore, further research is needed to understand the impacts of leptospirosis on threatened and endangered marine mammals. Additionally, *Leptospira* in the environment may threaten the implementation and success of efforts to re-introduce endangered species. Leptospirosis was a serious threat to the re-introduction of the Eurasian beaver (*Caster fiber*), which was formerly extirpated in much of its range and recently reintroduced in several European nations such as Scotland. Infection with *Leptospira* sometimes result in fatal clinical disease in Eurasian beavers [192,231]. Due to the potential for fatal clinical disease, careful monitoring and surveillance were taken with the re-introduction effort [232,233,234,235,236]. Another such study investigating the re-introduction of the endangered water vole, *Arvicola amphibius*, in the UK found that 42.9% of re-introduced voles were infected with *Leptospira* only four months post-release [237]. While there is currently no indication of impacts on this specific population from this infection (although this may be due to lack of study), leptospirosis is an exposure-dependent disease and other co-occurring species may be vulnerable to the elevated levels of *Leptospira* resulting from the re-introduction of a new population of reservoir hosts. This is not an unlikely scenario as studies have shown that tropical regions have some of the greatest occurrences of highly endangered biodiversity and are frequently targeted for conservation efforts [238].

## 6. Leptospirosis in the Environment: Across the Urban–Rural Gradient

The prevalence, rate of transmission, and route of transmission of *Leptospira* is not uniform across the landscape. This is unsurprising as there are significant environmental differences across the urban–rural gradient, including to (but not limited to) differences in hydrology, soils, and animal life. In our review of the literature, discussion of environmental conditions associated with leptospirosis tended to revolve around discussion of urbanization; however, studies found equivalent rates of leptospirosis regardless of urbanization [1,54,132,239]. Ultimately, we found that leptospirosis is not a disease that can be adequately described through rate of urbanization. In fact, meta-analysis of decades of leptospirosis research shows that morbidity of the disease is not significantly associated with urbanization and is greater in rural and tropical regions [1]. In contrast, there is significant evidence of greater severity of leptospirosis and mortality associated with urbanization [1,61,239]. While we found no clear unifying trend that explains these patterns of leptospirosis morbidity or mortality across urban–rural gradients, clear trends emerged in many regions along the urban–rural gradient. As a result, we suspect that the trends along the urban–rural gradient in a given region are indicative of routes of transmission, contributors of environmental persistence, and the most likely risks of spillover from environmental sources. By this, we mean that leptospirosis is likely very regionally specific; thus, understanding the disease in a region will elucidate the dominant factors associated with the persistence and transmission of *Leptospira*. By studying such trends along the urban–rural gradient, opportunities for the management of the disease are likely to emerge.

### 6.1. Current Understanding

Leptospirosis is associated with urbanization in some regions. The association of urbanization and leptospirosis most likely results from environmental conditions that contribute to environmental persistence, increase the risk of spillover from environmental sources, and facilitate common routes of transmission. In one such study, the primary risk factors proved to be variation in rat populations and exposure to mud flows and flooding regardless of whether the rats or flooding occurred in an urban or rural environment [239]. In urban environments, high-density low-income housing is associated with the transmission of *Leptospira* as a result of the contamination of housing and soils by infected urine from rat infestations [3,128,130,131,240,241]. In regions with high populations of people and rats, there is a greater risk of leptospirosis infection among both animals and humans, as studies suggest a population-associated accumulation of disease load [68]. Essentially, leptospirosis rates should be greatest where there is a high population of infected individuals and a high population of susceptible individuals. This is one of the prevailing theories behind studies that suggest greater leptospirosis in urban regions [132].

Additionally, socioeconomically disadvantaged communities have disproportionate rates of leptospirosis [1,11,34,35,37,38]. In urban areas, socioeconomically disadvantaged communities often reside in high-density housing with conditions that facilitate the spread of leptospirosis by increasing the contact with environmental sources of the disease. These conditions include reduced rates of governmental sanitation interventions, trash accumulation, poor infrastructure, and contact with sewer water [8,35,38,52,239,240]. Low-income and socioeconomically disadvantaged communities receive reduced rates of governmental sanitation interventions such as trash pickup and pest control, which increases rodent populations that play a large role in urban leptospirosis [128,129,130,131,134,135,136,137,138,139,140]. The aging or impaired infrastructure commonly found in low-income urban communities results in increased contact with sewer water for residents. For example, many outdated urban stormwater drains connect with sewer systems. In urban areas, these outdated stormwater systems are most commonly found in historically marginalized and socioeconomically disadvantaged communities [242]. High percentages of impervious surfaces in urban watersheds increase the urban-heat effect and the amount of storm runoff. This results in stormwater surges filling sewers and causing aboveground overflows of sewage and stormwater that contribute to the transmission of pathogens [243,244]. This situation promotes unsanitary, wet soils that are prone to frequent flooding. Consequently, many urban areas are characterized by high-risk conditions following large precipitation events. In addition, low-income housing is often constructed on wet soils in flood-prone regions along streams and other waterways [36]. Residing in flood-risk regions with wet soils has been significantly linked to risk of leptospirosis infection and outbreak during mass precipitation and flooding events [1,8,36,50,53,245,246]. Residents are also at greater risk outside of these mass precipitation and flooding events since they have greater contact with the wet soils that appear to be linked with long-term *Leptospira* persistence and survival. Additionally, as people are most likely to recreate in streams and waterways close to home, residents may be spending significant time in and around contaminated waterways. Residents may be unaware of their risk of *Leptospira* exposure and infection, as low-income and socioeconomically disadvantaged communities often do not receive equitable public health outreach [247]. In addition, low-income and socioeconomically disadvantaged communities have been shown to face disproportionate impacts from floods and other precipitation-based natural disasters due to reduced or delayed governmental assistance, lack of resources, and inequitable access to medical care [248,249,250].

The disproportionate impacts of leptospirosis on socioeconomically disadvantaged communities are not limited to urban contexts and extend to rural communities [1,11,34,35,37,38]. As we mentioned earlier in the review, there is significant evidence that leptospirosis cases occur most commonly in rural tropical regions [1]. Through our review, we found that the greater morbidity of leptospirosis in tropical rural communities seems to be associated with greater local prevalence of the pathogen and increased contact with potential environmental sources. Tropical rural regions likely have greater leptospirosis prevalence due to favorable environmental conditions that facilitate the greater environmental presence of other zoonotic pathogens. The warmer temperatures and increased precipitation rates found in the tropics are favorable for zoonotic pathogens [251]. These climatic conditions facilitate soil dynamics that are favorable for the survival of other enteric zoonotic pathogens such as *E. coli* [252]. The greater abundance and biodiversity of wildlife in tropical regions then facilitate greater environmental transmission of zoonotic pathogens [253]. Thus, there is greater potential for zoonotic pathogens such as *Leptospira* to complete their lifecycles through environmental transmission and wildlife in tropical regions, resulting in potentially greater prevalence in tropical rural regions. In addition, livestock and agricultural practices in tropical regions have increased human–wildlife–livestock interactions [254]. This may also facilitate greater rates of leptospirosis in tropical and rural regions. Additionally, agriculture and livestock production are generally located in rural communities. Thus, there are usually greater numbers of agricultural and livestock workers in rural communities and, thus, a greater risk of zoonotic disease [255]. This close contact with contaminated soils, waters, and animals likely facilitates greater local prevalence of leptospirosis in rural regions.

The leptospirosis risks associated with flooding and contaminated stormwaters are just as prevalent in rural communities as in urban [239]. In addition, inadequate sanitation interventions are also prevalent in socioeconomically disadvantaged rural communities and result in trash accumulation, contact with sewage, and other environmental exposures [239]. Similar to the impacts that outdated combined sewer overflows have on leptospirosis risk, inadequate and failing infrastructure increase the potential of leptospirosis exposure in rural communities, such as in the overflow of sewage lagoons and other livestock waste management solutions [256,257,258,259,260]. Similar to socioeconomically disadvantaged communities in urban settings, the impacts of leptospirosis on individuals have disproportionate impacts in rural areas. Lack of resources, quality medical care, and reduced or delayed governmental assistance can tremendously increase the burden and cycles of poverty [248,249,250,261].

### 6.2. Future Directions

As discussed, increased risk of leptospirosis along the urban–rural gradient often stems from a combination of factors associated with poverty, including lack of resources, poor infrastructure, and reduced governmental sanitation interventions. Additionally, increased risk of leptospirosis along the urban–rural gradient may also result from land management decisions that alter the hydrology and soil dynamics of waterways in tropical regions [262,263]. Alterations to hydrologic and biogeochemical conditions has been shown to determine virulence of disease, although this has yet to be studied in leptospirosis [264,265].

Since many of the increased risks associated with increased rates of leptospirosis are related to infrastructure and land management decisions, we suggest that by studying trends along the urban–rural gradient, opportunities for reducing the risk of leptospirosis outbreaks are likely to emerge alongside the increased understanding of where risk associated with environmental conditions is most severe. For example, prevalence within cattle herds increases along the urban–rural gradient as the proximity to the city increases [180]. The mechanism behind this trend is uncertain at this time, so further study is needed to understand the transmission cycles among domestic animals. There also appears to be a prevalence trend of leptospirosis among small mammal populations that occurs along the urban–rural gradient with different prevalence of infection between urban, suburban, and rural ecosystems [245]. The mechanisms behind this are also unknown. This highlights the lack of understanding on the cycles of *Leptospira* transmission in wildlife. Without a clear understanding of the routes of transmission, contributors of environmental persistence, and the most likely risks of spillover from environmental sources, efforts to control the disease are unlikely to be as effective as they could be. Due to this, it is imperative to study the dynamics of the disease across the urban–rural gradient alongside studies into the environmental persistence, survival, and reproduction of *Leptospira*. Targeted public health outreach to improve communities’ ability to participate in reducing disproportionate exposure to leptospirosis requires that we discover more about the environmental persistence and survival of *Leptospira* [266].

## 7. Conclusions

In this review, we discussed the current understanding of leptospirosis in the environment and highlighted future directions of study that we suggest will greatly assist in disease surveillance and management. Although the need for expanded active surveillance, treatment, and epidemiological transmission studies is not new, increasing pressures on natural systems are accelerating the frequency and severity of zoonotic disease outbreaks [34]. As climate change increases the variability, frequency, and severity of precipitation and storm events, the conditions required for outbreaks of zoonotic diseases such as leptospirosis will occur more frequently and in greater severity [267]. Urbanization concentrates human populations in high-density environments where outbreaks of disease are more common and have greater transmission rates, while the spread of urbanization globally places more and more people in increased contact with wildlife and zoonotic pathogens [268]. As these pressures increase the occurrence of *Leptospira* transmission, the impacts of this disease will disproportionately impact people of lower socioeconomic status without access to timely, quality healthcare, and will further exacerbate systemic cycles of poverty in these communities [3,4,34]. In addition, water resources are under more and more demand for use for consumption and recreation; as this demand grows, the risk of leptospirosis infection and outbreak will increase as well [16,31]. As a result, it is imperative that further study into the prevalence and transmission of *Leptospira* in the environment is conducted, with specific attention being paid to objectives that improve disease surveillance and management efforts.

## Figures and Tables

**Table 1:** Environmental studies of *Leptospira* survival in water. Only studies conducted in the field were included in the table.

Study Location	*Leptospira* Studied	Sampling Success	Survival	pH	Temperature	Citation
Tawain	Pathogenic and intermediate	NA ^1^	7 days	7–8	0–30 °C	Ryu et al., 1966 [76]
Philippines and Japan	Multiple pathogenic and intermediate	31/73	0 days	NA ^2^	NA ^2^	Saito et al., 2013 [56]
New Caledonia	*L. interrogans* Pyrogenes	0/10	0 days	NA ^2^	~28 °C	Thibeaux et al., 2017 [55]

^1^ Involved inoculated samples being placed into environment. ^2^ Not measured.

## Data Availability

The data presented in this study are openly available from the databases listed at: https://www.lib.auburn.edu/find/bytitle.php, accessed on 1 September 2022.
